# Phytochemical Analysis, In Vitro Biological Activities, and Computer-Aided Analysis of *Potentilla nepalensis* Hook Compounds as Potential Melanoma Inhibitors Based on Molecular Docking, MD Simulations, and ADMET

**DOI:** 10.3390/molecules28135108

**Published:** 2023-06-29

**Authors:** Subhash Sharma, Vikas Kumar, Muhammad Yaseen, Amr S. Abouzied, Abgeena Arshad, Mashooq Ahmad Bhat, Ahmed M. Naglah, Chirag N. Patel, Prasanth Kumar Sivakumar, Anuradha Sourirajan, Adnan Shahzad, Kamal Dev

**Affiliations:** 1 Faculty of Applied Sciences and Biotechnology, Shoolini University, P.O. Box 9, Head Post Office, Solan 173212, India; subhashsharma1434@gmail.com (S.S.); asourirajan@gmail.com (A.S.); 2 Department of Biotechnology, University Institute of Biotechnology, Chandigarh University, Gharuan, Mohali 140413, India; vikaskmr59@gmail.com; 3 Institute of Chemical Sciences, University of Swat, Charbagh, Swat 19130, Pakistan; adnanshahzad09@gmail.com; 4 Department of Pharmaceutical Chemistry, National Organization for Drug Control and Research (NODCAR), Giza 12311, Egypt; as.ibrahim@uoh.edu.sa; 5 Asian Medical Institute, Kant 725013, Kyrgyzstan; geenarsh238@gmail.com; 6 Department of Pharmaceutical Chemistry, College of Pharmacy, King Saud University, Riyadh 11451, Saudi Arabia; 7 Department of Botany, Bioinformatics and Climate Change Impacts Management, University School of Science, Gujarat University, Ahmedabad 380009, India; chiragpatel269@gmail.com (C.N.P.); prasanthbioinformatics@gmail.com (P.K.S.); 8 Biotechnology Research Center, Technology Innovation Institute, Abu Dhabi 9639, United Arab Emirates; 9 Department of Pharmacology and Toxicology, Wright State University, Dayton, OH 4543, USA

**Keywords:** *Potentilla nepalensis*, biological activities, antioxidant activity, anticancer activity, GC-MS analysis, molecular docking, MD simulations, toxicity prediction

## Abstract

*Potentilla nepalensis* Hook is a perennial Himalayan medicinal herb of the Rosaceae family. The present study aimed to evaluate biological activities such as the antioxidant, antibacterial, and anticancer activities of roots and shoots of *P. nepalensis* and its synergistic antibacterial activity with antibacterial drugs. Folin–Ciocalteau and aluminium chloride methods were used for the calculation of total phenolic (TPC) and flavonoid content (TFC). A DPPH radical scavenging assay and broth dilution method were used for the determination of the antioxidant and antibacterial activity of the root and shoot extracts of *P. nepalensis*. Cytotoxic activity was determined using a colorimetric MTT assay. Further, phytochemical characterization of the root and shoot extracts was performed using the Gas chromatography–mass spectrophotometry (GC-MS) method. The TPC and TFC were found to be higher in the methanolic root extract of *P. nepalensis*. The methanolic shoot extract of *P. nepalensis* showed good antioxidant activity, while then-hexane root extract of *P. nepalensis* showed strong cytotoxic activity against tested SK-MEL-28 cells. Subsequently, in silico molecular docking studies of the identified bioactive compounds predicted potential anticancer properties. This study can lead to the production of new herbal medicines for various diseases employing *P. nepalensis*, leading to the creation of new medications.

## 1. Introduction

Throughout human history, medicinal plants have been employed in traditional medicine and are regarded as a source of healthy human habitation. Different plant sections, such as the roots, leaves, stems, bark, fruits, and seeds, have been used to boost immunity and prevent a number of ailments [1]. Plants produce secondary metabolites (small organic chemicals) that are mostly needed for reproduction and defense mechanisms against bacteria, fungi, viruses, vertebrates, etc., but are not essential for their regular growth or development. These goods have strong potential for use as drugs. The WHO has identified over 21,000 plants that are widely used for medical reasons around the world. About 2500 species have been found in India, and biopharmaceutical companies exploit over 150 of them commercially on a sizable scale as conventional medicine. India, which holds the title of “the botanical garden of the globe”, is the country that produces the most medicinal plants. Interesting options exist in traditional medicine to fight MDR (multidrug resistance). Herbal medications exhibit a diverse range of biological activity, making them effective tools for treating disease. Combining dietary and medicinal approaches could result in a potent method for managing a variety of disorders [2].

The Indian Himalayan region is one of the richest reservoirs of biodiversity in the world. This region is rich in medicinal herbs and plants, which are used by the local people for various medicinal purposes at home as well as being the basic constituents of the medicines and other products available on the market [3]. *Potentilla nepalensis* is a medicinal herb of the Rosaceae family found in the Indian Himalayan region, especially in the North-Western Himalayas region. The genus *Potentilla* is well known for its pharmacological activities and bioactive compounds. The existence of several phytochemicals originating from various plant parts of the *Potentilla* species can be used to explain their pharmacological effects. In many cultures around the world, *Potentilla* extracts have been used to cure a variety of ailments. Compounds extracted from various portions of *Potentilla* species plants have been shown to have anti-inflammatory, anti-hyperglycemic, anticancer, and anti-ulcerogenic activities [4]. *Potentilla* species plants have been found to be high in phenolics, flavonoids, and terpenoids, as well as having strong antioxidant and antibacterial effects. Some *Potentilla* species have been employed in traditional remedies for their anti-diabetic, anti-diarrheal, anti-viral, anti-inflammatory, wound-healing, and anticancer properties. Root extracts from some *Potentilla* species have also been used to treat viral infections in Tibetan traditional medicine [1]. The roots of *P. nepalensis* are traditionally used for thr treatment of headaches, cold, asthma, dysentery, skin diseases, and various other ailments [5].

Melanoma is a type of skin cancer that is highly aggressive and caused by the malignant proliferation of melanocytes [6,7]. The incidence of melanoma is increasing globally at a rate of approximately 3–7% per year, making it a significant public health concern [8]. Timely detection of melanoma usually results in successful surgical treatment [9]. However, advanced or metastatic melanoma does not respond well to current treatments, which include targeted therapy and immunotherapy due to their high cost and tumor resistance development [10,11]. Therefore, there is a need for novel, effective, and easily accessible therapeutic approaches for melanoma that overcome these limitations. Glycogen synthase kinase 3 (GSK3) is a serine/threonine protein kinase found in all cells, regulating multiple cellular processes such as glycogen metabolism, signal transduction, cell cycle regulation, and cell proliferation [12,13,14]. GSK3 plays a crucial role in regulating the oxidative stress response associated with cancer occurrence and progression. Recent studies [15,16] have demonstrated that GSK3 overexpression makes breast cancer cells more sensitive to chemotherapeutic drugs and facilitates elastin-induced ferroptosis. A report from John et al. [17] showed that low constitutive levels of GSK3 signaling control both N-cadherin expression and the formation of focal adhesion complexes, which in turn contribute to the oncogenic nature of melanoma. GSK-3 regulates melanoma proliferation and shape via phosphorylation and elevated PAX3 levels [18].

Molecular docking analysis is a significant tool that predicts molecule activity and affinity by depicting the binding position of molecules to protein targets [19,20]. This study aims to investigate the antioxidant potential, anticancer activities, and total phenolic and flavonoid content of the methanolic and n-hexane extracts of the roots and shoots of *P. nepalensis*, a medicinal plant from the North-Western Himalayas region, using MTT assay against SKMEL-28 cells. In silico molecular docking, MD simulations, and toxicity prediction were performed to identify potential bioactive compounds with anticancer potential to discover and formulate new drug formulations.

## 2. Results

### 2.1. Quantification of Total Phenolic and Flavonoid Content

The data of the TPC and TFC of the roots and shoots extracts of *P. nepalensis* are shown in Table 1. A higher TPC (21.21 ± 0.54 mg/g GAE) and TFC (4.24 ± 0.17 mg/g RE) was observed in the methanolic extract of *P. nepalensis* roots as compared to that of P. nepalensis shoots.

### 2.2. In Vitro Antioxidant Activity

The DPPH scavenging activity of the *P. nepalensis* roots and shoots was found to be concentration-dependent and expressed in terms of IC_50_ value. Among all extracts, the methanolic extract of the *P. nepalensis* shoots showed high antioxidant activity with the smallest IC_50_ value being 12.83 *±* 0.35µg/mL, followed by the methanolic root extract (IC_50_-23.5 *±* 0.92 µg/mL). The standard L-ascorbic acid was found to show strong antioxidant activity (IC_50_-5.86 *±* 0.13µg/mL), while the n-hexane extract of both the roots and shoots of *P. nepalensis* exhibited low antioxidant activity (Table 1).

### 2.3. Cytotoxic Activity of Roots and Shoots of P. nepalensis

**Figure 1:**
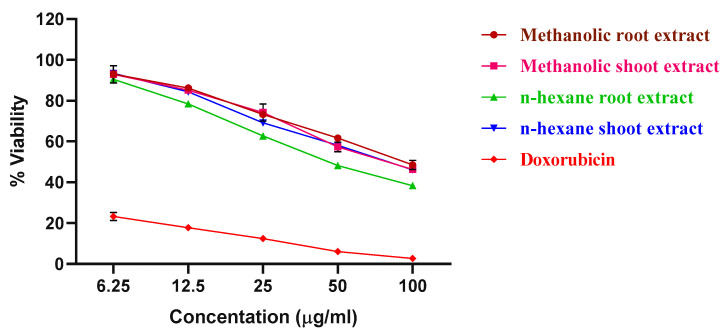
Concentration–effect of the roots and shoots of *P. nepalensis* in SK-MEL-28 cells obtained using the MTT assay (48 h exposure). Values were expressed as mean ± S.D. of three independent experiments.

**Figure 2:**
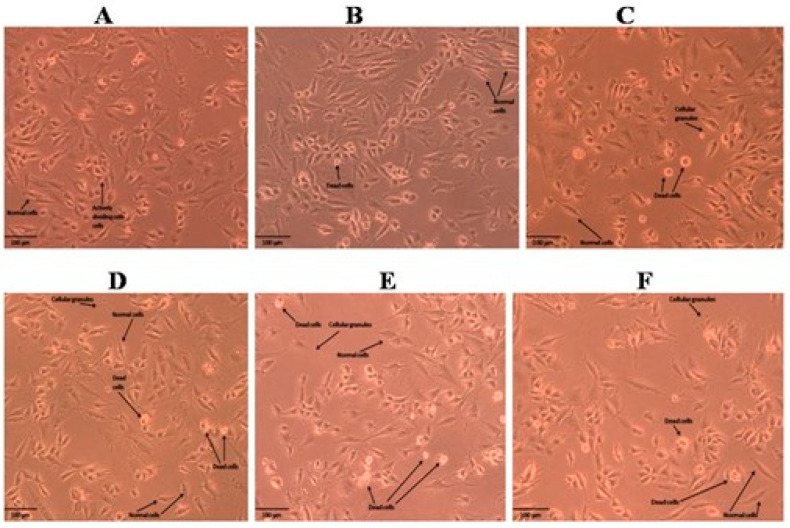
Cell morphology of SKMEL-28 cells after treatment with different concentrations of methanolic extract of roots of *P. nepalensis*. (**A**) Control; (**B**) 6.25 µg/mL; (**C**)12.5 µg/mL; (**D**) 25 µg/mL; (**E**) 50 µg/mL; (**F**) 100 µg/mL. Concentration-dependent reduction in cell number was observed in SKMEL-28 cells when administered with different concentrations of the extract. Cells showed alterations in morphology, such as rounding up and detachment from the surface, indicating the probable progression to apoptosis. The presence of cellular debris can also be seen, indicating the damage to cell structure.

**Figure 3:**
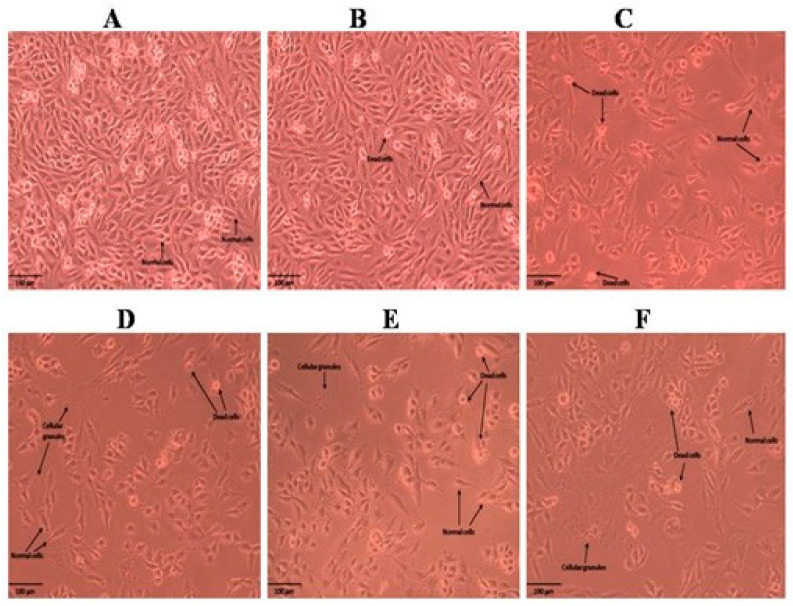
Cell morphology of SKMEL-28 cells after treatment with different concentrations of methanolic extract of shoots of *P. nepalensis*. (**A**) Control; (**B**) 6.25 µg/mL; (**C**)12.5 µg/mL; (**D**) 25 µg/mL; (**E**) 50 µg/mL; (**F**) 100 µg/mL. Concentration-dependent reduction in cell number was observed in SKMEL-28 cells when administered with different concentrations of the extract. Cells showed alterations in morphology, such as rounding up and detachment from the surface, indicating the probable progression to apoptosis. The presence of cellular debris can also be seen, indicating the damage to cell structure.

**Figure 4:**
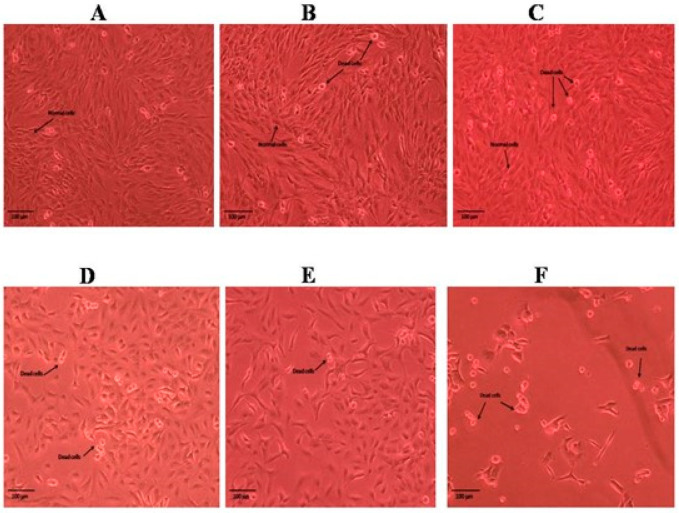
Cell morphology of SKMEL-28 cells after treatment with different concentrations of n-hexane extract of roots of *P. nepalensis*. (**A**) Control; (**B**) 6.25 µg/mL; (**C**)12.5 µg/mL; (**D**) 25 µg/mL; (**E**) 50 µg/mL; (**F**) 100 µg/mL. Concentration-dependent reduction in cell number was observed in SKMEL-28 cells when administered with different concentrations of the extract. Cells showed alterations in morphology, such as rounding up and detachment from the surface, indicating the probable progression to apoptosis. The presence of cellular debris can also be seen, indicating the damage to cell structure.

**Figure 5:**
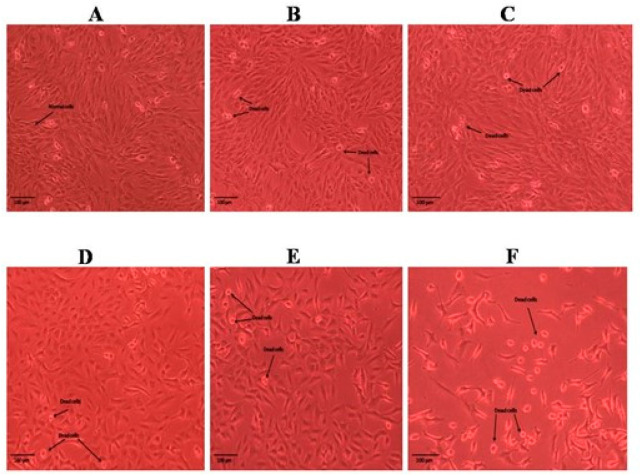
Cell morphology of SKMEL-28 cells after treatment with different concentrations of n-hexane extract of shoots of *P. nepalensis*. (**A**) Control; (**B**) 6.25 µg/mL; (**C**)12.5 µg/mL; (**D**) 25 µg/mL; (**E**) 50 µg/mL; (**F**) 100 µg/mL. Concentration-dependent reduction in cell number was observed in SKMEL-28 cells when administered with different concentrations of the extract. Cells showed alterations in morphology, such as rounding up and detachment from the surface, indicating the probable progression to apoptosis. The presence of cellular debris can also be seen, indicating the damage to cell structure.

The results obtained for the MTT assay of the methanolic and n-hexane extracts of the roots and shoots of *P. nepalensis* are shown in Figure 1. Among all the extracts of roots and shoots, the n-hexane extract of the *P. nepalensis* roots showed comparatively high cytotoxic activity (IC_50_-65.37 *±* 2.27 µg mL^−^^1^), followed by the n-hexane extract of the *P. nepalensis* shoots (IC_50_-81.97 *±* 2.75 µg mL^−^^1^) and methanolic shoot extract (IC_50_-82.74 *±* 1.54 µg mL^−^^1^) against tested SK-MEL-28 cells. The methanolic root extract was found to have the least cytotoxic effect (IC_50_-88.65 *±* 3.71 µg mL^−^^1^). Doxorubicin was used as positive control showing IC_50_-4.55 *±* 0.18 µg mL^−^^1^ against SK-MEL-28 cells. Both root and shoot samples elicited a concentration-dependent reduction in cell viability as indicated by the reduction in cell numbers from the Inverted phase-contrast microscopy images (Figure 2, Figure 3, Figure 4 and Figure 5). In addition to this, alterations in cell morphology, such as cell rounding up and cell fragmentation from the control group of cells, can be observed. All these together support the reduction in cell viability as indicated by the MTT assay.

### 2.4. Identification of Major Phytocompounds of Methanolic and n-Hexane Extracts of Roots and Shoots of P. nepalensis via GC-MS Profiling

**Figure 6:**
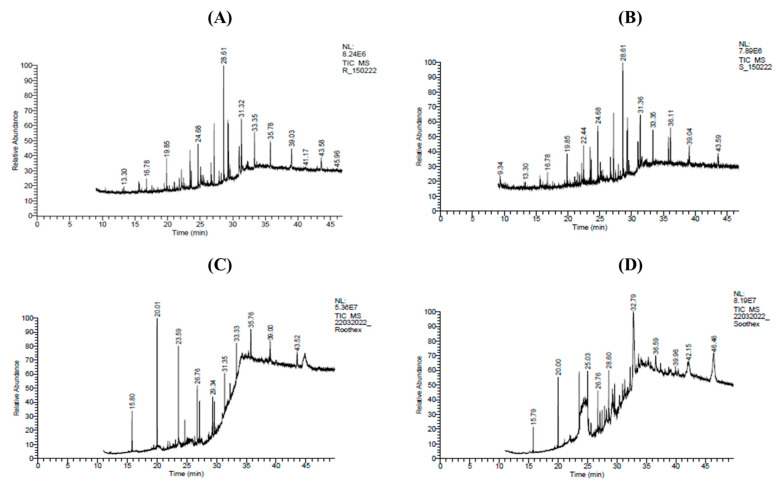
GC-MS chromatogram of roots and shoots of *P. nepalensis*: (**A**) methanolic root extract; (**B**) methanolic shoot extract; (**C**) n-hexane root extract; (**D**) n-hexane shoot extract. *X*-axis represents the time in minutes and, *Y*-axis represents the relative abundance of the phytocompound.

The GC-MS chromatograms of the methanolic and n-hexane extracts of the roots and shoots from *P. nepalensis* revealed the presence of 10 compounds each (Figure 6A–D). The major phytocompounds reported in the GC-MS chromatograms of the methanolic root extract of *P. nepalensis* were tetradecanoic acid, 10,13-dimethyl-, methyl ester (12.64%), and Heptadecanoic acid, 16-methyl-, methyl ester (10.58%) (Table 2); while the methanolic shoot extracts of P. nepalensis showed the presence of Hexadecanoic acid, and methyl ester (11.82%),1,1,1,5,7,7,7-Heptamethyl-3,3-bis(trimethylsiloxy)tetrasiloxane (8.58%) (Table 3). GC-MS analysis of the n-hexane root extract of *P. nepalensis* showed the presence of Trichloromethyl 9-anthracenecarbodithioate (22.90%), Heptane,3,3-dimethyl-(17.71%), and Hexadecanene (12.28%) (Table 4); while the n-hexane shoot extract of P. nepalensis showed the presence of Benzene, 1,3,5-tri-tert-butyl-(23.19%), 1,1,1,3,5,5,5-Heptamethyltrisiloxane (23.19%), and 4H-1-Benzopyran-2-carboxylic acid, 5-amino-6-hydroxy-4-oxo-, ethyl ester (17.32%) as major phytocompounds (Table 5).

### 2.5. Molecular Docking Analysis

**Figure 7:**
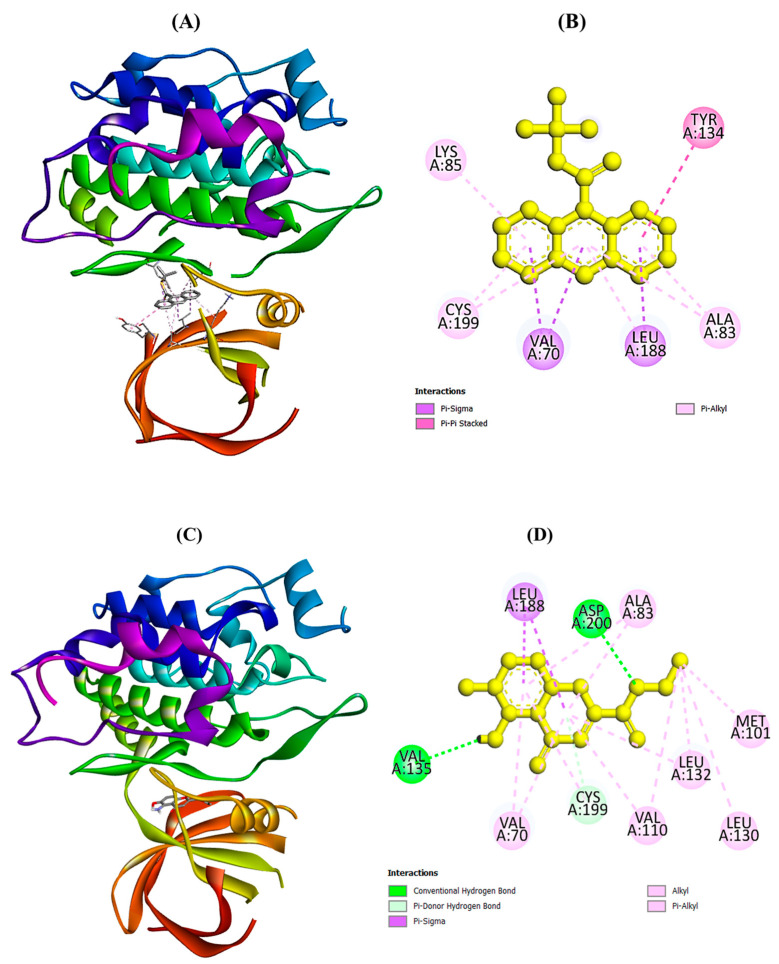
Docked pose of best docked phytocompounds from *P. nepalensis* against target protein (PDB ID: 5K5N): (**A**) 3D interactions of trichloromethyl 9-anthracenecarbodithioate with interacting amino acids of 5K5N; (**B**) 2D interactions of trichloromethyl 9-anthracenecarbodithioate with interacting amino acids of 5K5N; (**C**) 3D interactions of 4H-1-Benzopyran-2-carboxylic acid, 5-amino-6-hydroxy-4-oxo-, ethyl ester with interacting amino acids of 5K5N; (**D**) 2D interactions of 4H-1-Benzopyran-2-carboxylic acid, 5-amino-6-hydroxy-4-oxo-, ethyl ester with interacting amino acids of 5K5N.

The binding energies of selected phytocompounds of *P. nepalensis* with target proteins using the Glide (grid-based ligand docking) program are summarized in Table 6. Trichloromethyl 9-anthracenecarbodithioate (−8.9 kcal/mol) and 4H-1-Benzopyran-2-carboxylic acid, 5-amino-6-hydroxy-4-oxo-, ethyl ester (−7.4 kcal/mol) have shown the highest binding affinity among all selected phytocompounds. However, encorafenib (drug) was found to a show binding affinity of -8.6 kcal/mol with the selected target protein. Binding interactions of selected phytocompounds of *P. nepalensis* with interacting amino acids were analyzed using the Discovery Studio (DS) visualizer. Figure 7A–D showed the binding interactions of the best docked phytocompounds, viz., trichloromethyl 9-anthracenecarbodithioate and 4H-1-Benzopyran-2-carboxylic acid, 5-amino-6-hydroxy-4-oxo-, ethyl ester with amino acids of the selected target proteins (PDB ID: 5K5N).

### 2.6. MD Simulations Study

**Figure 8:**
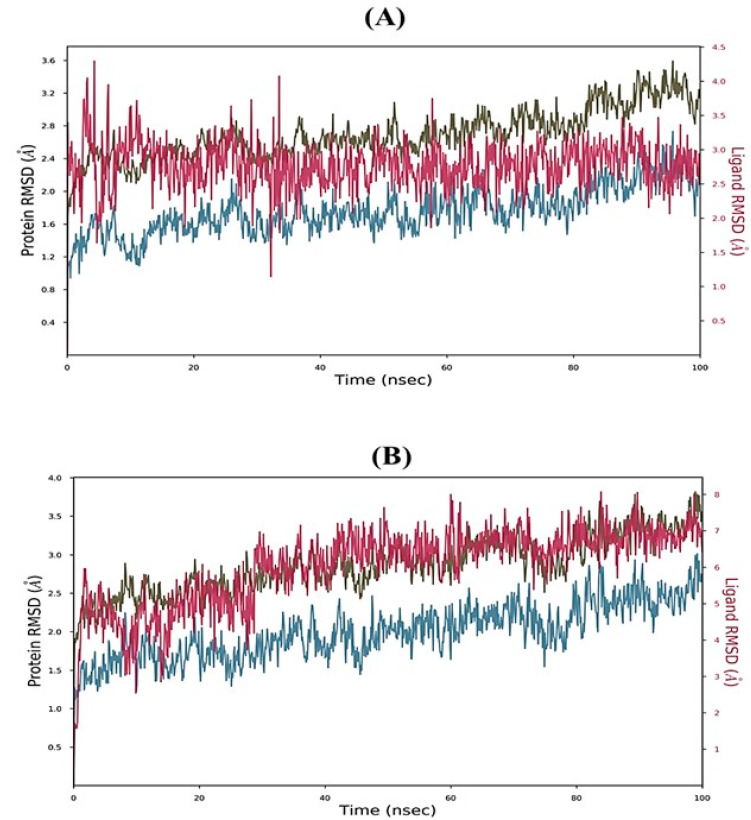
MD simulation of protein–ligand interaction root-mean-square deviation (RMSD) profiles of (**A**) trichloromethyl 9-anthracenecarbodithioate-5K5N complex and (**B**) 4H-1-Benzopyran-2-carboxylic acid, 5-amino-6-hydroxy-4-oxo-, ethyl ester-5K5N complex. Color legends: Ca (blue color), side chains (green color), heavy atoms (yellow color), ligand with protein (dark pink color), ligand with ligand (pink color).

MD simulations were conducted for 100 ns using the Academic version of the Desmond programme version 2.0 (Schrödinger LLC, New York, NY, USA) to evaluate the overall stability and flexibility of the ligand–protein complexes. Two phytochemicals that exhibited strong binding interactions with the 5K5N receptor protein were selected for the MD simulations. RMSD (root-mean-square deviation) was used to assess the fluctuations of the ligand within the active site of the receptor protein based on the MD trajectories. The RMSD values over time for the Cα atoms of the protein–ligand complex are presented in Figure 8. Both complexes remained stable during the MD simulations, as indicated by the protein backbone RMSD values hovering around 4.0 Å and well under 8 Å for the trichloromethyl 9-anthracenecarbodithioate (Figure 8A), and 4H-1-benzopyran-2-carboxylic acid, 5-amino-6-hydroxy-4-oxo-, ethyl ester-5K5N (Figure 8B) complexes, respectively.

**Figure 9:**
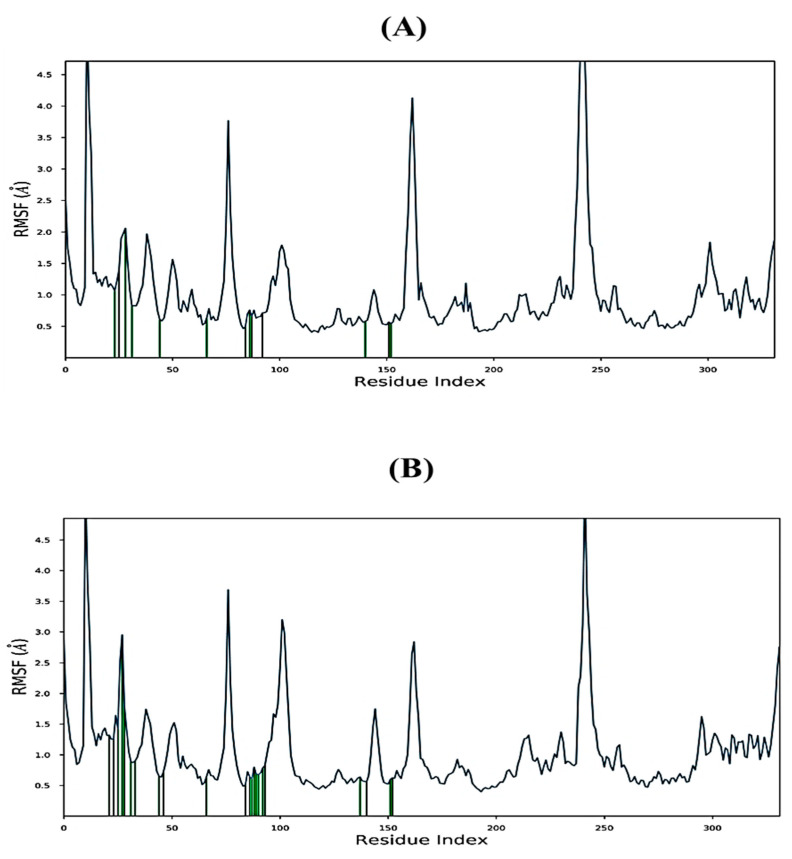
Analysis of RMSF trajectories over the 100 ns MD simulation for (**A**) trichloromethyl 9-anthracenecarbodithioate-5K5N complex and (**B**) 4H-1-Benzopyran-2-carboxylic acid, 5-amino-6-hydroxy-4-oxo-, ethyl ester-5K5N complex. Protein residues that interact with ligand are marked with green-colored vertical bars.

The root-mean-square fluctuation (RMSF) is a useful tool for identifying changes in protein structure, as shown in Figure 9A,B. The amino acids of the 5K5N proteins with trichloromethyl 9-anthracenecarbodithioate displayed fluctuations ranging between 0.5–5.0 Å (Figure 9A), while the fluctuations of the amino acids of the 5K5N proteins with 4H-1-Benzopyran-2-carboxylic acid, 5-amino-6-hydroxy-4-oxo-, ethyl ester were represented in Figure 9B. In the RMSF plot, peaks correspond to the portions of the receptor proteins that exhibited maximum fluctuations during the MD simulation. Generally, protein tails, identified through the N and C terminals, display higher fluctuations than rigid structures such as α-helices and β-strands. These structured regions are stiffer than unstructured regions, hence showing minimal fluctuations. The highest RMSF values obtained during the MD simulation for both protein–ligand complexes were 4.6 Å in Glu249 (Figure 5A,B), likely due to its high flexibility resulting from the formation of a β-turn in the protein structure at that position [37].

**Figure 10:**
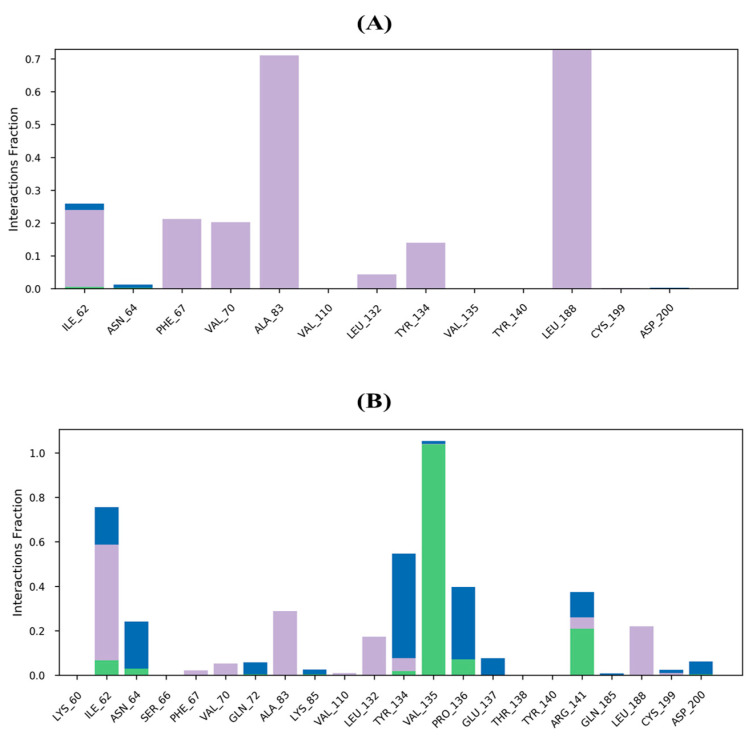
Protein–ligand contact histograms (hydrogen bonding, hydrophobic interactions, ionic interactions, and water bridge of (**A**) trichloromethyl 9-anthracenecarbodithioate-5K5N complex and (**B**) 4H-1-Benzopyran-2-carboxylic acid, 5-amino-6-hydroxy-4-oxo-, ethyl ester-5K5N complex. Different color in bars indicates different interaction as hydrogen bond (green), hydrophobic contacts (purple) and water-bridge (blue).

Protein–ligand interactions offer valuable insights into simulation techniques, conformational stability, and correlated effects. These interactions can be classified into four main subtypes—Hydrogen Bonds, Hydrophobic, Ionic, and Water Bridges—each with explicit subtypes that can be studied using the ‘Simulation Interactions Diagram’ board. The stacked bar outlines used to represent the interactions are standardized, and an estimation of 0.8 indicates that during 80% of the simulation time the interaction is sustained. However, values over 1.0 are possible as some protein build-ups may create multiple contacts of the same subtype with the ligand. In the complex consisting of trichloromethyl 9-anthracenecarbodithioate-5K5N, hydrophobic interactions with the ligand were found to occur with Ile 62, Phe 67, Val 70, Ala 83, Leu 132, Tyr 134, and Leu 188 (Figure 10A). Amino acids Val 135 and Arg 141 were the most important for hydrogen bonding in the 4H-1-Benzopyran-2-carboxylic acid, 5-amino-6-hydroxy-4-oxo-, ethyl ester-5K5N complex, while amino acids Ala 83, leu 132, and Leu 188 were the most significant for hydrophobic interactions (Figure 10B).

### 2.7. Assessment of Drug Likeness and Toxicity Prediction

Table 7 represents the drug likeness and toxicity prediction of the best docked phytocompounds of *P. nepalensis*. Lipinski’s rule of five and ADMET prediction were used to evaluate the pharmacokinetic and toxicity properties of the top-ranked compounds. The druggability and toxicity parameters were achieved for both top-ranked phytocompounds, except for carcinogenicity. Trichloromethyl 9-anthracenecarbodithioate was predicted to be carcinogenic, with an LD_50_ of 493 mg/kg (Class IV). In contrast, 4H-1-Benzopyran-2-carboxylic acid, 5-amino-6-hydroxy-4-oxo-, ethyl ester had an LD_50_ of 100 mg/kg (Class III) (Table 7).

## 3. Discussion

Plant phenolics and flavonoids are currently a major study focus since they are assumed to be responsible for the bulk of biological activities of plants, such as their anti-inflammatory, antibacterial, antiviral, antioxidant, and anticancer capabilities. Medicinal plants have played an important role in the discovery of approximately 50% of anticancer drugs. In our study, we have observed high phenolic and flavonoid content in the methanolic extract of roots and shoots of P. nepalensis, whereas n-hexane extracts of roots and shoots of *P. nepalensis* showed lower amounts of phenolic and flavonoid content, which may be due to the higher solubility of phenolics and flavonoids in methanol extract as compared to that of n-hexane extract [38]. Tomczyk et al. [39] reported a higher TPC (73.9 *±* 3.7 mg GAE/g dw) in the aqueous extract of areal parts of P. nepalensis, while the TFC in the aqueous extract (2.1 *±* 0.5 mg QE/g dw) was found to be comparable with our results [39]. This variation in TPC content can be related to differences in geographical regions, solvent preference, plant age, and plant part selection [40,41,42]. Recently, a study by Sharma et al. [43] reported a higher phenolic (21.21 *±* 0.54 mg g^−1^ GAE) and flavonoid content in wild grown plants (4.24 *±* 0.17 mg g^−1^ RE) as compared to that of in vitro propagated plants (TPC-3.55 *±* 0.72 mg g^−1^ GAE; TFC-0.33 *±* 0.07 mg g^−1^ RE).

Free radicals, which can harm cell membranes and other structures, are unstable molecules produced by the oxidation process in the human body. These free radicals have been linked to several diseases, including heart disease and various cancers. Antioxidants, which are chemicals that scavenge and battle free radicals and are present in medicinal plants, may reduce the risk of a variety of diseases, including heart disease and several types of cancer. We examined the in vitro antioxidant potential of methanolic and n-hexane extracts of *P. nepalensis* roots and shoots while taking the positive effects of antioxidants into consideration. The antioxidant activity of methanolic root extract of *P. nepalensis* was also reported by Sharma et al. [43]. Low IC_50_ values of methanolic extracts indicating strong free radical scavenging or antioxidant capabilities were observed in our study. The strong antioxidant potential in methanolic root and shoot extracts as compared to n-hexane extracts can be attributed to the higher amount of phenolic and flavonoid content [44].

It is well recognized that medicinal plants contain a variety of chemicals with the ability to prevent or treat a wide range of illnesses. As cancer is a serious issue for public health, we preferred using an MTT assay for the methanolic and n-hexane extracts of P. nepalensis roots and shoots against SKMEL-28 cells. The n-hexane extract of the P. nepalensis roots was found to be most effective against SKMEL-28 cells with the smallest IC_50_ value. Additionally, there is very little analytical data on the chemical makeup of *P. nepalensis*, and we used GC-MS profiling to analyze the methanolic and n-hexane extracts of the plant’s roots and shoots to determine their primary phytocompounds. The majority of phytocompounds identified via GC-MS profiling of the methanolic and n-hexane extracts of *P. nepalensis* roots and shoots have been reported for biological activities such as antimicrobial, antioxidant, and anticancer activities and have also been discovered in the plant extracts of some other therapeutic plants. The presence of these phytocompounds may be responsible for the medicinal properties of *P. nepalensis*.

Computational approaches are extremely valuable in pharmaceutical research since they aid in the discovery and development of new, promising medicines, particularly when used in conjunction with molecular docking techniques. These techniques have been used by several research teams to screen possible new chemicals against a variety of ailments. In silico pharmacokinetic, pharmacological, and toxicological performance have also been predicted using them. In the present study, we have utilized a molecular docking system to analyze the binding affinities of selected phytocompounds of *P. nepalensis* with amino acids of glycogen synthase kinase 3β (GSK3β) protein to check the overall stability and/or flexibility of the ligand–protein complexes as well as the drug likeness and toxicity prediction of the best docked phytocompounds of P. nepalensis. To our knowledge, this is the first report on the anticancer activity and GC-MS characterization of the shoots and roots of P. nepalensis. Molecular docking and MD simulation studies also support the anticancer activity of P. nepalensis. Further, analysis of the drug-like effects of selected compounds in this study displayed the potential of these compounds as anticancer drugs. The selected potential drug candidates were also found to fulfill the toxicity criteria. Overall, the selected phytochemicals are good potential drug candidates to treat myeloma in the future.

## 4. Materials and Methods

### 4.1. Chemicals and Reagents

The aluminum chloride, ascorbic acid, dimethyl sulfoxide (DMSO), 2,2- diphenyl-2-picrylhydrazyl (DPPH), ferric chloride, Folin–Ciocalteau reagent, gallic acid, n-hexane, methanol, rutin, and sodium nitrite (NaNO_2_) were purchased from Loba Chemie Pvt. Ltd., Mumbai, India. 3-(4,5-dimethyl-2-thiazolyl)-2,5-diphenyl-2H-tetrazolium bromide (MTT), Dulbecco’s modified eagle medium (DMEM), nutrient broth, nutrient agar, ampicillin, chloramphenicol, and resazurin were purchased from Himedia Laboratories Pvt. Limited, Mumbai, India. All the chemicals and reagents utilized in this study were of analytical grade.

### 4.2. Collection and Identification of Plant Material

*Potentilla nepalensis* plants collected during July to September 2020 from Kurri, Shimla, Himachal Pradesh, at a height of 2600 m above sea level were used as experimental material. The sample was validated by the Department of Forest Products, Dr. Y.S. Parmar University of Horticulture and Forestry, Nauni, Solan, Himachal Pradesh under voucher no 13925.

### 4.3. Preparation of Extracts of Roots and Shoots of P. nepalensis

The roots and all the above ground parts were harvested from *P. nepalensis* plants and cut into pieces. The cut pieces were washed with running tap water followed by distilled water and dried at 40 °C and converted to fine powder using an electric grinder. About 10 g of fine powder was added to 100 mL of 70% methanol and n-hexane in separate culture tubes and macerated for 24 h at 30 °C for extract preparation [31,32,33]. The filtrate was collected and evaporated at 40 °C. The dried crude extracts were stored in airtight bottles at 4 °C for further use.

### 4.4. Spectrophotometric Quantification of Total Phenolic (TPC) and Flavonoid Content (TFC)

The TPC and TFC of the methanolic and n-hexane extracts of roots and shoots of *P. nepalensis* was performed using the Folin–Ciocalteau (FC) reagent method [45,46,47] and aluminum chloride (AlCl_3_) method [43,48]. Standard curve of Gallic acid (5–80 µg/mL) and Rutin (5–80 µg/mL) was used for calculation of the TPC and TFC, respectively.

### 4.5. In Vitro Antioxidant Potential Using DPPH Radical Scavenging Assay

The DPPH radical scavenging method was used to assess the in vitro antioxidant capacity of the roots and shoots of *P. nepalensis* [49]. To 100 µL of varied final concentrations of extracts (5–20 µg/mL), 900 µL of 0.004% DPPH solution (*w*/*v*, in methanol) was added and allowed to react in the dark for 15 min. In the control reaction, 100 µL of methanol was added to 900 µL of 0.004% DPPH solution. After incubation, absorbance was measured at 517 nm using a UV-visible spectrophotometer. Ascorbic acid (5–20 µg/mL) was used as standard. The following equation was used to calculate the DPPH scavenging activity:

$$\mathrm{DPPH}\;\mathrm{radical}\;\mathrm{scavenging}=\frac{O.D.\;\left(Control\right)-O.D.\;\left(Standard/Test\right)}{O.D.\;\left(Control\right)}\times 100$$

where O.D. (control) is the absorbance of the control, and O.D. (standard/test) is the absorbance of the extract/standard. The IC_50_ value was calculated to express the free radical scavenging activity of extracts, showing the effective concentration of extract/standard used to scavenge 50% of DPPH radicals. The lower the IC_50_ value, the more the scavenging ability of the extract.

### 4.6. In Vitro Cytotoxic Activity of P. nepalensis Roots and Shoots

The cytotoxic properties of the extracts of the roots and shoots of *P. nepalensis* against myeloma (SK-MEL-28) cells were determined using the MTT assay. To begin with, the cells were obtained from culture flasks through trypsinization and transferred into 96-well microculture plates containing 100 μL aliquots of DMEM supplemented with 10% heat-inactivated fetal bovine serum, 1 mM sodium pyruvate, 2 mM L-glutamine, and 1% non-essential amino acids (100×). The cell density was adjusted to 2.5 × 10^3^ cells per well to ensure their exponential growth. The cells were allowed to settle and grow in a drug-free complete culture medium for 24 h, after which they were exposed to various dilutions of the test samples (6.25, 12.5, 25, 50, and 100 µg/mL) dissolved in DMSO for 48 h. The control wells were left untreated. Doxorubicin was used as a positive control. Next, the medium was replaced with an RPMI 1640 medium along with MTT solution in phosphate-buffered saline (PBS), and the mixture was incubated for another 2 h. The viable cells produced formazan product that was dissolved in DMSO (150 μL/well), and the optical densities were measured at 570 nm with a microplate reader, using the reference wavelength of 690 nm to correct for non-specific absorption. The percentage of viable cells was determined, and the IC_50_ values were calculated [50]. The IC_50_ values were calculated using the equation for slope (y = mx + C) obtained by plotting the absorbance of the different concentrations of the test/drug sample (6.25–100 µg/mL) in Microsoft Excel.

### 4.7. GC-MS Profiling of Root and Shoot Extracts of P. nepalensis to Identify Major Phytocompounds

The GC-MS analysis of the roots and shoots of *P. nepalensis* was conducted using a Thermo Fisher Scientific Gas Chromatograph equipped with a Tri Plus RSH Autosampler, GC trace-1300, and MS-TSQ Duo. The Thermo Fisher Scientific TG-5MS column was utilized, which measured 40 m in length, 0.15 mm in film, and 0.15 m in internal diameter. The method involved setting the first oven temperature to 80 °C, with a temperature increase of 8 °C/min and a 1-min hold period, followed by increasing the temperature to 150 °C, with a rate of 10 °C/min and a 6-min hold period. The total run time was 32 min, with a 1 µL sample volume injected using helium at a flow rate of 0.7 mL/min as the carrier gas. The MS was operated within the electron ionization (EI) mode, scanning within a 40–450 amu range with a mass spectrometer source temperature and transfer line temperature set at 230 °C and 250 °C, respectively, and an electron multiplier voltage of 1 kV. Mass spectra were interpreted using the NIST/EPA/NIH Mass Spectral Library Version 2.2, 2014, and fragmentation patterns were compared with the instrument database data for all constituents detected.

### 4.8. Ligand Preparation

The three-dimensional structures of identified phytochemicals and Encorafenib (drug) were obtained from Pubchem (https://pubchem.ncbi.nlm.nih.gov/, accessed on 1 March 2023) (Figure S1). Extracts containing phytocompounds above 10% were selected as ligands for docking studies. The ligands and drug were minimized using Chem3D structure software and converted to pdbqt format using Open Babel (http://openbabel.org/, accessed on 1 March 2023).

### 4.9. Retrieval and Preparation of Target Proteins

The three-dimensional structure of GSK-3 beta complexed with PF-04802367 (PDB ID: 5K5N) [51] (Figure S2) was downloaded from the protein data bank (https://www.rcsb.org/, accessed on 5 March 2023) and used Auto-Dock tool 1.5.6 for the preparation of the protein structure. The active site of the target protein was predicted based on the previously bounded native ligand [38], and the grid box parameters for 5K5N were set to a size of 37.05 Å × 23.225 Å × 23.35 Å (x, y, and z) with a center at coordinates 4.056 Å × 2.085 Å × 29.143 Å (x, y, and z).

### 4.10. Molecular Docking

A total of seven major phytocompounds of *P. nepalensis* were docked against GSK-3 beta protein using the AutoDock vina tool [52]. The top scoring phytocompounds were selected based on their binding energy with target protein receptors. The best pose based on binding energies for each ligand–protein interaction was further analyzed using the Discovery Studio (DS) visualizer (Accelrys, San Diego, CA, USA).

### 4.11. Molecular Dynamics Simulations

Desmond program version 2.0 (academic version) was used to investigate the stability of the receptor–ligand complexes formed via molecular docking [53,54]. The system was prepared using the TIP3P water model with a cubic periodic box incorporating Simple Point Charge (SPC) (10 Å × 10 Å × 10 Å) with Optimized Potentials for the Liquid Simulations (OPLS) all-atom force field 2005 [55]. Sodium ions were added to neutralize the system. The receptor–ligand complexes were subjected to energy minimization and pre-equilibration through several restricted steps. MD simulations were conducted with periodic boundary conditions using the OPLS 2005 force field parameters with a relaxation time of 1 ps at a constant temperature of 300 K and constant volume in the NPT ensemble system [56,57]. The Smooth Particle Mesh Ewald (PME) approach was used to analyze protein structures every 1 ns. The stability was calculated from an average structure obtained from the production phase of the MD simulation. The authors examined the structural changes of the receptor–ligand complexes using the histogram for torsional bonds, the radius of gyration (Rg), the root-mean-square deviation (RMSD), and the root-mean-square fluctuation (RMSF) to understand the dynamic role played by the complexes [58,59,60,61].

### 4.12. Evaluation of Drug-Likeness and ADME/Toxicity Properties

Lipinski’s rule (rule of five, RO5) was considered the primary factor for screening of the molecules, and it was evaluated using the SWISS ADME web server (http://www.swissadme.ch/, accessed on 10 March 2023). Further, the toxicity of selected compounds was analyzed using the Protox-II tool to ascertain their risk of druggability [62,63]. 

## 5. Conclusions

The present study provides evidence that *P. nepalensis* shoots and roots have antioxidant and anticancer properties. Various phytocompounds with known biological activity were detected via GC-MS profiling of *P. nepalensis*, further supporting the therapeutic significance of this Himalayan plant. In addition, molecular docking, MD simulation studies, and druggability analyses were performed on GSK-3 beta protein with the selected phytochemicals, trichloromethyl 9-anthracenecarbodithioate and 4H-1-Benzopyran-2-carboxylic acid, 5-amino-6-hydroxy-4-oxo, ethyl ester. Throughout the duration of the MD simulation, both phytocompounds stayed firmly connected to the target proteins, i.e., the GSK-3 beta protein. The drug-likeness and ADMET analyses of both of these drug candidates for the treatment of melanomas revealed their drug-like properties. Both phytochemicals were found to be non-toxic; therefore, their anticancer activities to treat or suppress melanoma could be the subject of future experimental studies.

## Figures and Tables

**Table 1:** TPC, TFC, and DPPH free radical scavenging activity of the methanolic and n-hexane extracts of the roots and shoots of *P. nepalensis*.

Plant Part	Solvent Used	TPC (mg g^−1^ GAE)	TFC (mg g^−1^ RE)	DPPH Activity IC_50_ (µg mL^−1^)
Roots	Methanol	21.21 ± 0.54	4.24 ± 0.17	23.5 ± 0.92
Shoots	Methanol	15.68 ± 2.79	2.58 ± 0.1	12.83 ± 0.35
Roots	n-Hexane	0.90 ± 0.19	0.06 ± 0.03	65.69 ± 0.77
Shoots	n-Hexane	1.59 ± 0.13	0.49 ± 0.06	74.93 ± 1.01
Ascorbic Acid	Methanol	-	-	5.86 ± 0.13

**Table 2:** Major phytocompounds identified in GC-MS profiling of methanolic extracts of roots of *P. nepalensis*.

RT (min)	Area%	Name of Phytocompound	Molecular Formula	Bioactivity Reported	References
28.61	12.64	Tetradecanoic acid, 10,13-dimethyl-, methyl ester	C_17_H_3_4O_2_	-	-
31.32	10.58	Heptadecanoic acid, 16-methyl-, methyl ester	C_19_H_38_O_2_	Effective against skin cancer	[21]
27.15	6.44	1,1,1,5,7,7,7-Heptamethyl-3,3-bis (trimethyl siloxy) tetrasiloxane	C_13_H_40_O_5_Si_6_	Anti-quorum sensing	[22,23]
29.26	6.34	Phthalic acid, butyl hept-4-yl ester	C_19_H_28_O_4_	-	-
29.35	5.87	1,1,1,3,5,5,7,7,7-Nonamethyl-3-(trimethyl siloxy) tetrasiloxane	C_12_H_36_O_4_Si_5_	-	-
24.68	4.95	Norepinephrine, (R)-, 4TMS derivative	C_20_H_43_NO_3_Si_4_	-	-
31.02	4.63	2-Isopropyl-6-phenylnicotinonitrile	C_15_H_14_N_2_	-	-
33.35	4.56	Cyclononasiloxane, octadecamethyl	C_18_H_54_O_9_Si_9_	antifungal, antibacterial and/or antiviral properties	[24,25]
39.03	4.23	Oxalic acid, 2TMS derivative	C_8_H_18_O_4_Si_2_	-	-
23.47	3.8	2-Tetradecanol	C_14_H_30_O	Ingredient in cosmetics such as cold creams for its emollient properties	[26]

**Table 3:** Major phytocompounds identified in GC-MS profiling of methanolic extracts of shoots of *P. nepalensis*.

RT (min)	Area%	Name of Phytocompound	Molecular Formula	Bioactivity Reported	Reference
28.61	11.82	Hexadecanoic acid, methyl ester	C_17_H_34_O_2_	Antimicrobial	[27]
31.36	8.58	1,1,1,5,7,7,7-Heptamethyl-3,3-bis(trimethylsiloxy)tetrasiloxane	C_13_H_40_O_5_Si_6_	Anti-quorum sensing	[22,23]
29.35	5.54	Oxalic acid, 2TMS derivative	C_8_H_18_O_4_Si_2_	-	-
24.68	5.4	Cyclooctasiloxane, hexadecamethyl-	C_16_H_48_O_8_Si_8_	-	-
36.11	5.11	3-Ethyl-7-hydroxyphthalide	C_10_H_10_O_3_	Antioxidant	[28]
29.26	4.7	Phthalic acid, butyl hex-3-yl ester	C_18_H_26_O_4_	Antimicrobial, insecticidal activity	[29]
33.35	4.36	1,1,1,3,5,5,7,7,7-Nonamethyl-3-(trimethylsiloxy)tetrasiloxane	C_12_H_36_O_4_Si_5_	-	-
31.02	4.09	1-Hexyl-1-nitrocyclohexane	C_12_H_23_NO_2_	Antimicrobial and anti-inflammatory	[30]
22.44	3.71	Isobutyl 4-hydroxybenzoate	C_11_H_14_O_3_	Antimicrobial activity	[31]
39.04	3.55	Octanoic acid, 5-(acetyloxy)-, methyl ester	C_11_H_20_O_4_	-	-

**Table 4:** Major phytocompounds identified in GC-MS profiling of n-hexane extracts of roots of *P. nepalensis*.

RT (min)	Area%	Name of Phytocompound	Molecular Formula	Bioactivity Reported	Reference
44.85	22.9	Trichloromethyl 9-anthracenecarbodithioate	C_16_H_9_Cl_3_S_2_	-	-
20.01	17.71	Heptane, 3,3-dimethyl-	C_9_H_20_	-	-
23.59	12.28	Hexadecane	C_16_H_34_	Antifungal, Antibacterial, antioxidant activity	[32]
39	7.01	1,1,1,5,7,7,7-Heptamethyl-3, 3-bis(trimethylsiloxy)tetrasiloxane	C_13_H_40_O_5_Si_6_	Anti-quorum sensing	[22,23]
15.8	5.57	Dodecane	C_12_H_26_	Antifungal activity	[32]
26.76	4.99	Eicosane	C_20_H_42_	Antimicrobial activity against clinical pathogens	[33]
29.34	4.79	1,1,1,3,5,5,7,7,7-Nonamethyl-3-(trimethylsiloxy) tetrasiloxane	C_12_H_36_O_4_Si_5_	-	[34]
33.33	4.68	Propanoic acid, 2-oxo-3-(trimethylsilyl)-, trimethylsilyl ester	C_9_H_20_O_3_Si_2_	-	-
43.52	4.3	1,1,1,3,5,7,7,7-Octamethyl-3,5-bis(trimethylsiloxy) tetrasiloxane	C_14_H_42_O_5_Si_6_	-	[35]
11	2	2,4,6,9-Dehydroadamantane	C_10_H_12_	-	-

**Table 5:** Major phytocompounds identified in GC-MS profiling of n-hexane extracts of shoots of *P. nepalensis*.

RT (min)	Area%	Name of Phytocompound	Molecular Formula	Bioactivity Reported	Reference
25.03	23.19	Benzene, 1,3,5-tri-tert-butyl-	C_18_H_30_	-	-
32.79	23.19	1,1,1,3,5,5,5-Heptamethyltrisiloxane	C_7_H_22_O_2_Si_3_	Anti-inflammatory and antimicrobial properties	[36]
46.46	17.32	4H-1-Benzopyran-2-carboxylic acid, 5-amino-6-hydroxy-4-oxo-, ethyl ester	C_12_H_11_NO_5_	-	-
42.15	10.13	1-Propene, 3-methoxy-	C_4_H_8_O	-	-
29.24	8.05	Phthalic acid, butyl hept-4-yl ester	C_19_H_28_O_4_	-	[29]
20	3.14	Heptane, 3,3-dimethyl-	C_9_H_20_	-	-
36.59	2.71	N- (Methyl sulfonyl)-N, O-bis (trimethyl silyl) hydroxylamine	C_7_H_21_NO_3_SSi_2_	-	-
28.6	2.3	Dodecanoic acid, 2-methyl-	C_13_H_26_O_2_	-	-
38.8	2.27	2-Acetyl-3-ethylpyrazine	C_8_H_10_N_2_O	-	-
26.76	1.87	Eicosane	C_20_H_42_	Antimicrobial activity against food-borne pathogens	[33]

**Table 6:** Binding energy of docked phytocompounds from *P. nepalensis* against targeted amino acids. Binding energy was expressed in terms of Kcal/mol. The amino acids showing H-bonding with ligands were represented in red color fonts.

Sr. No.	Phytocompounds	PubChem ID	PDB ID: 5K5N	
			Binding Energy (Kcal/mol)	Interacting Amino Acids
1	Trichloromethyl 9-anthracenecarbodithioate	613595	−8.9	Val A:70, Ala A:83, Lys A:85, Tyr A:134, Leu A:188, Cys A:199
2	4H-1-Benzopyran-2-carboxylic acid, 5-amino-6-hydroxy-4-oxo-, ethyl ester	619354	−7.4	Val A:70, Ala A:83, Met A:101, Val A:110, Leu A:130, Leu A:132, Val A:135, Leu A:188, Cys A:199, Asp A:200
3	Benzene, 1,3,5-tri-tert-butyl-	15089	−7.1	Ile A:62, Phe A:67, Val A:70, Leu A:132, Cys A:199
4	Heptane, 3,3-dimethyl-	520991	−4.6	Val A:70, Ala A:83, Lys A:85, A:132, Tyr A:134, Leu A:188, Cys A:199
5	Hexadecane	11006	−5.1	Ile A:62, Val A:70, Ala A:83, Lys A:85, Leu A:132, Tyr A:134, Leu A:188, Cys A:199
6	1,1,1,3,5,5,5-Heptamethyltrisiloxane	6327366	−3.6	-
7	1-Propene, 3-methoxy-	69392	−2.8	Tyr A71, Ile A: 84
8	Encorafenib (drug)	50922675	−8.6	Gly A:65, Phe A:67, Lys A:183, Asp A:200

**Table 7:** Drug likeness and toxicity prediction of best docked phytocompounds of *P. nepalensis*.

Compound	cLogP	n_rot_	MW	HBD	HBA	Lipinski Rule	Hepato-Toxicity	Immuno-Genicity	Carcino-Genicity	Cyto- Toxicity	LD_50_ (mg/kg)
Trichloromethyl 9-anthracenecarbodithioate	3.29	3	371.73	0	0	Yes	No	No	Yes	No	493 mg/kg (Class IV)
4H-1-Benzopyran-2-carboxylic acid, 5-amino-6-hydroxy-4-oxo-, ethyl ester	1.89	3	249.22	2	5	Yes	No	No	No	No	100 mg/kg (Class III)

clogP (<5)—Measure of molecular hydrophobicity; n_rot_ (<5)—Number of rotatable bonds; MW (<500Da)—Molecular weight; HBA (<10)—H-bond acceptor; HBD (<5)—H-bond donor; LD_50—_Lethal dose.

## Data Availability

Not applicable.

## Supplementary Materials

The following supporting information can be downloaded at: https://www.mdpi.com/article/10.3390/molecules28135108/s1, Figure S1: Chemical structures of identified compounds used for molecular docking.; Figure S2: Three-dimensional structure of target protein, Glycogen Synthase Kinase-3(PDB ID: 5K5N).
