# Supplementary material for: Phytochemical Analysis, In Vitro Biological Activities, and Computer-Aided Analysis of Potentilla nepalensis Hook Compounds as Potential Melanoma Inhibitors Based on Molecular Docking, MD Simulations, and ADMET
Source: Molecules. 2023 Jun 29;28(13):5108. doi: 10.3390/molecules28135108 (PMC10343834; doi:10.3390/molecules28135108)
Supplement: Supplementary file 1 [file molecules-28-05108-s001.zip › molecules-2426251-supplementary.pdf]

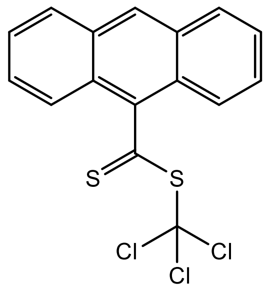

Trichloromethyl 9-anthracenecarbodithioate

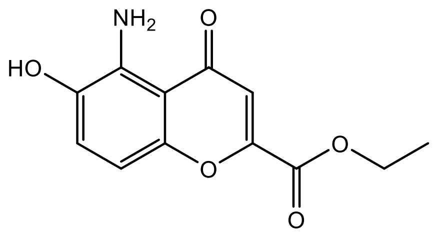

4H-1-Benzopyran-2-carboxylic acid, 5-amino-6-hydroxy-4-oxo-, ethyl ester

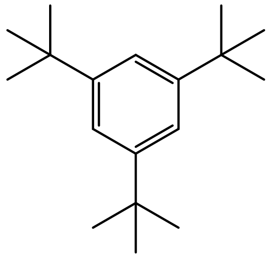

Benzene, 1,3,5-tri-tert-butyl-

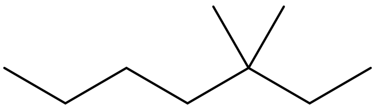

Heptane, 3,3-dimethyl-

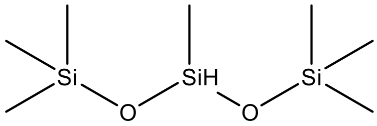

1,1,1,3,5,5,5-Heptamethyltrisiloxane

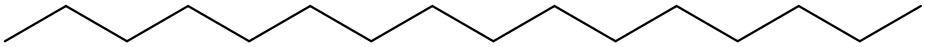

Hexadecane

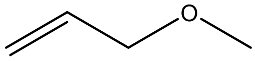

1-Propene, 3-methoxy-

Figure S1. Chemical structures of identified compounds used for molecular docking.

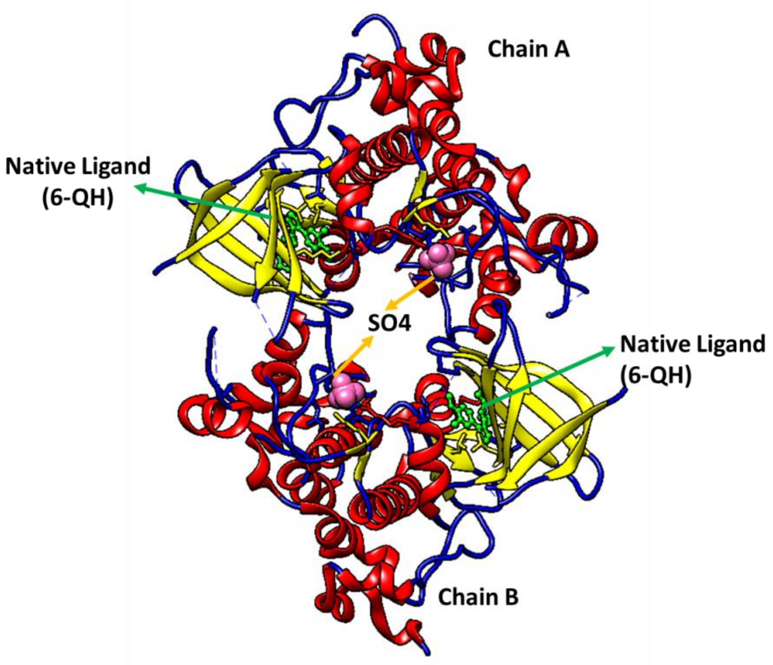

Figure S2. Three-dimensional structure of target protein, Glycogen Synthase Kinase-3(PDB ID: 5K5N).The alpha helices were denoted in red, beta-sheets in yellow and turns and loops in blue color. The proteins were in a complex with residues, SO4 and 6-QH.
